# Universal, selective and indicated interventions for supporting mental health at the workplace: an umbrella review of meta-analyses

**DOI:** 10.1136/oemed-2022-108698

**Published:** 2023-02-24

**Authors:** Clara Miguel, Arpana Amarnath, Aemal Akhtar, Aiysha Malik, Gergő Baranyi, Corrado Barbui, Eirini Karyotaki, Pim Cuijpers

**Affiliations:** 1 Department of Clinical, Neuro and Developmental Psychology, Vrije Universiteit Amsterdam, Amsterdam, The Netherlands; 2 School of Psychology, University of New South Wales, Sydney, New South Wales, Australia; 3 Division of Insurance Medicine, Department of Clinical Neuroscience, Karolinska Institutet, Stockholm, Sweden; 4 Department of Mental Health and Substance Use, World Health Organization, Geneva, Switzerland; 5 Society and Health, The University of Edinburgh, Edinburgh, UK; 6 Department of Neuroscience, Biomedicine and Movement Sciences, University of Verona, Verona, Italy; 7 WHO Collaborating Centre for Research and Dissemination of Psychological Interventions, Vrije Universiteit Amsterdam, Amsterdam, The Netherlands

**Keywords:** meta-analysis, occupational health, mental health

## Abstract

The current umbrella review aimed to assess and summarise evidence on universal, selective and indicated interventions for mental health at the workplace. This umbrella review forms one of the evidence reviews which were commissioned by the WHO to develop global guidelines on mental health at work. We conducted systematic searches in five bibliographic databases (PubMed, Embase, PsycINFO, Cochrane and Global Medicus Index) and included meta-analyses of randomised trials examining psychosocial, physical activity and lifestyle interventions delivered to all general workers (universal interventions), at-risk workers (selective interventions) and workers already experiencing symptoms of mental disorders (indicated interventions). We included outcomes from seven domains: symptoms of mental health conditions, positive mental health, quality of life, work-related outcomes, substance use, suicide-related outcomes and potential adverse effects. We identified 16 meta-analyses producing 66 pooled effect sizes of the examined interventions, mostly on symptoms of mental health conditions (n=43 pooled effect sizes) (eg, burnout, insomnia, stress) and positive mental health (n=15) (eg, well-being). Most of the evidence on universal, selective and indicated interventions was focused on psychosocial interventions, showing small to moderate effects across the various outcomes. Certainty levels according to GRADE (Grading of Recommendations Assessment, Development and Evaluation) were low to very low in almost all of the examined outcomes. The results of existing meta-analyses are promising for the use of preventative and early treatment interventions in the workplace. However, the quality and certainty of the evidence were very modest, and further research on the effectiveness of these interventions is warranted.

**WHAT IS ALREADY KNOWN ON THIS TOPIC**

- Previous meta-analyses have examined the effectiveness of workplace mental health interventions. However, these meta-analyses usually focus on a specific population, intervention or outcome, and a comprehensive view of this research field is needed.

**WHAT THIS STUDY ADDS**

- This umbrella review summarised the effects of universal, selective and indicated preventative strategies to support mental health at the workplace. The results of the included meta-analyses are promising for the use of preventative and early treatment interventions in the workplace. Furthermore, our review revealed important knowledge gaps and highlighted the need for further research.

**HOW THIS STUDY MIGHT AFFECT RESEARCH, PRACTICE OR POLICY**

- This umbrella review supported the WHO Guideline Development Group to develop global guidelines on mental health at work. Therefore, it will have a significant impact on the research, practice and policy in the field of occupational health.

Globally, it has been estimated that more than 970 million individuals experienced a mental disorder in 2019, with 80.6% of the burden of disease occurring among working-age individuals.^1^ Common mental disorders, such as depression or anxiety, are one of the leading causes of long-term disability worldwide while generating a serious impact on economies.^2 3^ In the absence of scaled up treatment, depression and anxiety have been estimated to cost the global economy US$1 trillion each year.^4^ This is largely due to the productivity losses derived from sickness absence, presenteeism and turnover.^5–7^ Further, the WHO defines mental health as not just the absence of a mental disorder, but rather a state of mental well-being in which individuals are able to cope with normal life stressors, realise their own abilities, be able to learn and work fruitfully and contribute to their communities.^8 9^ In this line, positive mental health and well-being have been associated with better social relationships, physical health, job performance and job satisfaction, among others, as well as with impacts on the productivity of organisations.^10–12^

The workplace can offer a unique setting for delivering interventions to preventing and supporting mental health conditions. Given that working-age adults spend a large proportion of their time at work, implementing interventions at the workplace could increase the access and uptake of evidence-based interventions. Different strategies can be delivered at the workplace for mental health promotion, prevention and early treatment, depending on the focus of the delivered intervention. In the mental health field, these strategies are often classified as universal, selective and indicated interventions.^9 13^ Universal interventions are addressed at all general workers, regardless of the risk level of the individuals, with the expectation to provide some benefits to all the receiving population. Selective interventions are targeted to subgroups of populations that are at a higher risk for developing a mental disorder. Some occupations have been associated with an increased risk for mental health problems because the workplace presents a greater adversity by its design. For instance, health workers are a particularly vulnerable group because of chronic exposure to work stressors, showing a high risk for burnout, depression or suicidal behaviors.^14–16^ Similar concerns have been raised for other at-risk occupations, such as emergency workers^17^ or humanitarian workers.^18^ At a later stage of the mental health intervention spectrum, indicated prevention and early treatment strategies are addressed to individuals who are identified as having symptoms from mental health disorders (eg, workers who are experiencing burnout, high levels of stress or depressive symptoms). In all these strategies, the emphasis is set on the superiority of benefits over harms, taking costs into account as well.^19^

In addition, it is important to consider a broad spectrum of outcomes when examining the effects of such interventions. Considering the WHO definition of mental health,^8 9^ outcomes should go beyond only examining symptoms of mental health conditions and should include also positive mental health and outcomes related to functioning (eg, quality of life, functioning or work-related outcomes). In the workplace setting, outcomes such as work effectiveness or job satisfaction are especially relevant when assessing the overall effectiveness of interventions.

A growing body of research has examined the effectiveness of workplace interventions for preventing and protecting mental health, while such interventions are starting to be used routinely in organisations. Most meta-analyses on workplace interventions are focused on a specific target population (eg, physicians),^20^ intervention type (eg, mindfulness-based),^21^ delivery format (eg, e-health)^22^ or one specific outcome (eg, burnout)^23^ making it difficult to obtain a comprehensive overview of the effectiveness of these interventions across all levels of prevention and early treatment strategies. The rapid increase of research in the field and the large number of highly specialised meta-analyses highlight the need for a higher level of synthesis. Umbrella reviews, which are systematic reviews of systematic reviews, offer the opportunity to systematically present an overview of a research field and identify uncertainties and knowledge gaps.^24 25^

Therefore, we conducted an umbrella review with the aim of providing an evidence-based overview of universal, selective and indicated interventions for mental health at the workplace. For this, we systematically reviewed meta-analyses of randomised trials examining major types of workplace interventions delivered directly to the individuals (individual-level), namely psychosocial, physical activity and lifestyle interventions. The effects of these interventions were reviewed separately for each focus—universal, selective and indicated strategies—and summarising their effects on a wide range of outcomes, including mental health symptoms, positive mental health, quality of life or functioning and work-related outcomes (such as productivity, absence and work effectiveness).

## Methods

As part of the systematic literature searches to support the development of the WHO Guidelines for Mental Health at Work,^8^ we conducted an umbrella review on preventive and early treatment interventions for protecting mental health in workers. We aimed to systematically collect and review the effectiveness of universal, selective and indicated interventions on mental health symptoms, positive mental health, quality of life and work-related outcomes.

### Identification and selection of studies

Systematic literature searches were performed in PubMed (18 November 2020), PsycINFO (25 November 2020), Embase (27 January 2021) and Cochrane (27 January 2021). In April 2021, these searches were supplemented by an additional search in Global Medicus Index (12 April 2021) aiming to identify records from non-Western contexts, as well as an update of the original searches in PubMed (12 April 2021), which is the database that provided the largest number of relevant hits. In line with the WHO guideline methodology, indicating that evidence obtained for the development of guidelines should be as recent as possible,^26^ we limited the searches to studies published within the previous 5 years (which was since 1 January 2015). The full search strings for PubMed are provided in the online supplemental file. Two reviewers (CM, AAm) screened titles and abstracts independently and assessed the full text of any potentially eligible study. Disagreements were solved through consensus or by consulting with a third senior reviewer (PC).

10.1136/oemed-2022-108698.supp1 Supplementary data

The following inclusion criteria were used:

- *Major type of intervention based on the prevention and early treatment spectrum*: (1) Universal interventions (ie, addressed to workers who are not at an increased risk for mental health disorders and who are not selected based on a screening for mental health), (2) Selective interventions (ie, addressed to workers who are at an increased risk for mental health disorders due to the nature of their work, which was restricted to as healthcare, emergency or humanitarian workers in the context of the WHO guidelines^8^ and (3) Indicated interventions (ie, addressed to workers with elevated mental health symptoms, who are selected based on a mental health screening as part of the trial).
- *Specific subtypes of interventions based on their content or focus*: Interventions had to be delivered directly to (and for the direct benefit of) the individuals (ie, individual-level interventions) and could include the following categories: (1) psychosocial, including psychological interventions, (2) physical activity or (3) lifestyle (eg, diet for health promotion). Any treatment delivery format (individual, group, self-help, etc) was included.
- *Design of included studies*: The studies included in the meta-analysis had to be (or at least the vast majority of them; ie, >75%) randomised controlled trials (RCTs). We included meta-analyses with less than 75% of RCTs if the results for RCTs were reported separately.
- *Outcomes*: Symptoms of mental health conditions (eg, depressive symptoms, stress), positive mental health (eg, well-being, resilience), quality of life, work-related outcomes, substance use, suicidal behaviours and potential adverse effects of the intervention (eg, deterioration). A panel of WHO guideline development experts classified the outcomes as ‘critical’ and ‘important’ for each type of intervention (an overview of this classification is available in the online supplemental file).

We excluded interventions specifically administered to military personnel as this was outside the scope of the WHO guidelines.^8^ We also excluded organizational-level interventions, the training aimed at improving managers’ or workers’ mental health literacy, return-to-work and gaining employment programmes, as they were part of separate reviews contributing to the WHO guidelines.^8^ When multiple meta-analyses overlapped completely in a research question (ie, evaluated the same type of interventions, in the same population, and reported the same outcomes), we selected one meta-analysis based on recency, broadness and quality of the review as assessed with AMSTAR-2 (A MeaSurement Tool to Assess systematic Reviews-2).

### Data extraction

Two researchers (CM, AAm) independently extracted the following data from the meta-analyses: target of the intervention (ie, universal, selective, indicated), main types of interventions (ie, psychosocial, lifestyle, physical activity), details about the participants and data involving the effects of the interventions: outcome domain (eg, quality of life) and instrument, standardised mean difference (SMD) and its 95% CI, number of trials included in each analysis (*k*), p value of the SMD and the heterogeneity statistic *I* ^2^, with its 95% CI. When the 95% CI of the *I* ^2^ was not available, we calculated it using the value of χ^2^ and df with the Heterogi module in STATA SE (V.16.1 for Mac). When the 95% CI of an SMD was not available, we reported the p value. We extracted outcomes when a minimum of *k*=2 trials were pooled, with the exception of an outcome with critical importance according to the panel of experts (ie, suicide), for which we presented results derived from only one trial.

### Quality of the included reviews

The quality of the included meta-analyses was assessed using AMSTAR-2.^27^ AMSTAR-2 critically appraises core methodological characteristics of systematic reviews in 16 items (online supplemental file). Each item is assessed as positive (Yes) or negative (No), with some of them including a partially positive answer (Partial Yes). Two independent reviewers (CM, AAm) performed the ratings, and disagreements were solved by discussion or consultation with a third reviewer (PC).

### Certainty of the evidence

The certainty of the evidence was assessed using the Grading of Recommendations Assessment, Development and Evaluation (GRADE) system,^28^ according to the following five factors: risk of bias in the primary studies, inconsistency, indirectness, imprecision and other considerations (eg, risk of publication bias). The evaluation of these factors resulted in four levels of confidence, ranging from very low (the true effect is likely to be substantially different from the estimated effect) to high confidence (very confident that the true effect is similar to the estimated effect) (online supplemental file).

### Integration of findings

We classified all the identified meta-analyses based on the predefined main intervention types: (1) universal interventions, (2) selective interventions and (3) indicated interventions. We included meta-analyses that were completely focused on one of the types of interventions (eg, a meta-analysis completely focused on indicated interventions for workers with depressive symptoms), as well as broader meta-analyses that included mixed types of interventions and populations (eg, including universal interventions and selective interventions) when separate effect size data were specifically available for each type of intervention. Outcomes were classified into seven domains: symptoms of mental health conditions, positive mental health, quality of life, work-related outcomes, substance use, suicidal behaviours and adverse effects.

## Results

### Selection and inclusion of meta-analyses

**Figure 1:**
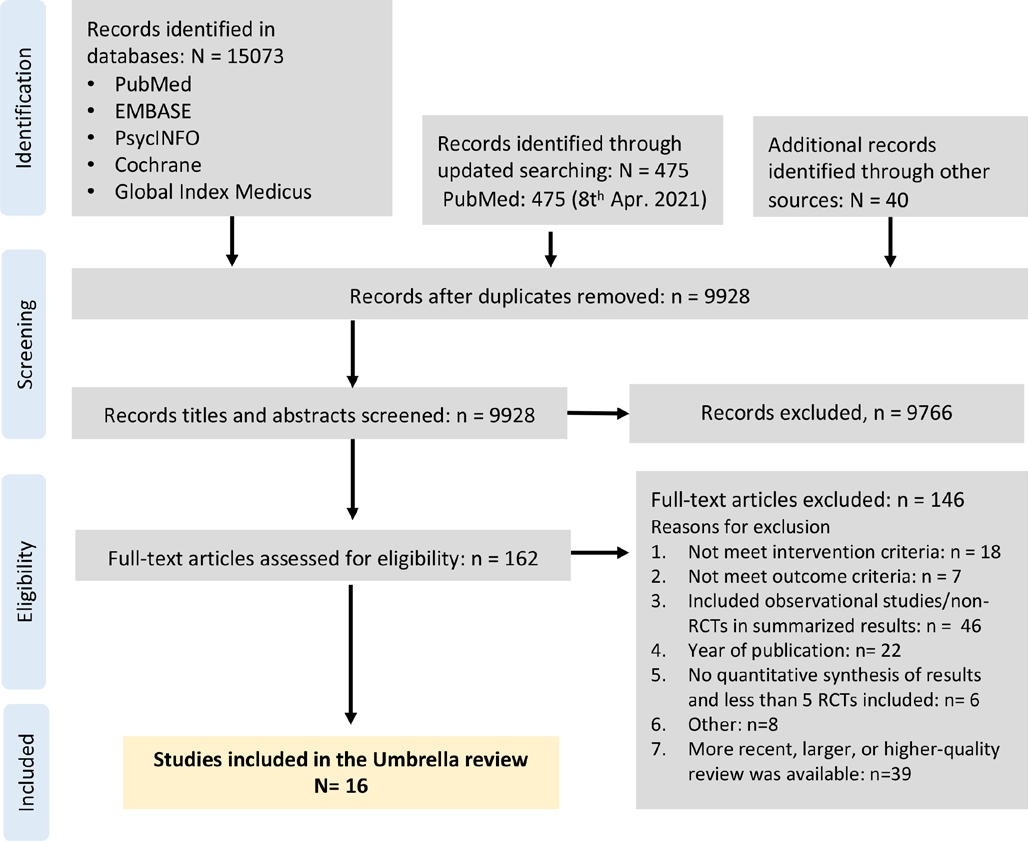
PRISMA flow chart describing the inclusion process.

A total of 15 588 records were identified, and 9928 titles and abstracts were screened after removal of duplicates. We retrieved 162 full-text articles and excluded 9766. The PRISMA (Preferred Reporting Items for Systematic Reviews and Meta-Analyses) flow chart describing the inclusion process, with reasons for exclusion, is presented in figure 1. A total of 16 meta-analyses met the inclusion criteria. The references of the included meta-analyses are presented in the online supplemental file 1.

### Characteristics of included meta-analyses

The main characteristics of the 16 meta-analyses are summarised in table 1. The total number of primary studies included in the reviews ranged from 6 to 119. The sample sizes varied, with the largest meta-analysis including a total of 10 232 participants.

**Table 1:** Selected characteristics of the 16 included meta-analyses

Study	Interventions	Aim	Population details	Content of interventions	Included studies	No of participants
Carolan, 2017^22^	1) Universal 3) Indicated	Identify the effectiveness of occupational digital mental health interventions in enhancing employee psychological well-being and increasing work effectiveness.	1) General working population from mixed occupations 3) Workers from mixed occupations with symptoms of depression, stress and insomnia	*Psychosocial (delivered through e-health):* Various internet-delivered psychological interventions, including CBT-based, stress and coping, mindfulness social cognitive theory, problem-solving training, positive psychology, and acceptance and commitment therapy.	21 RCTs	5260
Fendel, 2021^21^	2) Selective	Evaluate the effectiveness of mindfulness-based interventions in reducing burnout and stress among physicians.	Healthcare (physicians)	*Psychosocial:* Mindfulness-based interventions.	25 studies (6 RCTs)	326
Guillaumie, 2017^38^	2) Selective	Review the scientific literature on the effects of mindfulness on nurses and nursing practices.	Healthcare (nurses)	*Psychosocial:* Mindfulness-based interventions.	32 studies (16 RCTs)	875
Kunzler, 2020^36^	2) Selective	Assess the effects of interventions to foster resilience in healthcare professionals, that is, healthcare staff delivering direct medical care (eg, nurses, physicians, hospital personnel) and allied healthcare staff (eg, social workers, psychologists).	Healthcare (nurses, physicians, social workers, psychologists, etc)	*Psychosocial:* Psychological interventions for resilience (mindfulness, CBT, attention and interpretation therapy, stress inoculation, etc).	44 RCTs	6892
Maricuţoiu, 2016^23^	1) Universal 3) Indicated	Assess the effectiveness of controlled interventions on reducing employees’ burnout.	1) General working population from mixed occupations 3) Workers from mixed occupations with elevated symptoms of burnout	*Psychosocial:* CBT, relaxation, interpersonal, soft skills, hard skills, etc.	47 studies (34 RCTs)	2335
Melnyk, 2020^35^	2) Selective	Focus on randomised controlled trials (RCTs) with physicians and nurses that tested interventions designed to improve their mental health, well-being, physical health, and lifestyle behaviours.	Healthcare (nurses and physicians)	*Mixed (psychosocial, physical activity, lifestyle):* Mindfulness, lifestyle behavioural interventions, physical activity and healthy eating, stress-reduction interventions and CBT.	29 RCTs (17 in MA)	2708
Nigatu, 2019^40^	3) Indicated	Conduct a systematic review and meta-analysis on the effectiveness of indicated interventions for reducing depressive symptoms in the workplace.	Workers from mixed occupations with elevated depressive symptoms	*Psychosocial:* CBT, mental health literacy, psychoeducation, stress management, problem-solving, etc.	16 RCTs	2522
Oakman, 2018^31^	1) Universal	Analyse whether workplace interventions positively impact work ability.	General working population from mixed occupations	*Mixed (physical activity, lifestyle)*: Exercise programmes and education of individuals on healthy behaviours or coping strategies.	17 RCTs (13 in MA, 9 including*)	1502
Petrie, 2019^37^	2) Selective	Assess which, if any, interventions are effective at reducing or preventing symptoms of common mental health disorders or suicidality in physicians.	Healthcare (physicians)	*Psychosocial:* Psychological interventions (CBT, mindfulness, supportive, coping).	8 (7 RCTs)	1023
Phillips, 2019^34^	1) Universal 3) Indicated	Investigate the effectiveness of occupational e-mental health interventions aimed at stress, depression, anxiety, burnout, insomnia, mindfulness, well-being, and alcohol misuse.	1) General working population from mixed occupations 3) Workers from mixed occupations with symptoms of mental health conditions (depression, stress, anxiety)	*Psychosocial (delivered through e-health):* CBT, normative personalised feedback, mindfulness, psychoeducation, cognitive training, problem-solving training, positive psychology, etc.	50 RCTs (34 in MA)	10 232
Sakuraya, 2020^30^	1) Universal	Conduct a systematic review and meta-analysis of RCTs to improve SWB, including evaluative, hedonic and eudaemonic well-being, and mental components of QoL of the working population.	Workers from mixed occupations	*Mixed (psychosocial, physical activity, lifestyle):* Physical activity, psychological (mindfulness, CBT, meaning-centred, resilience), environmental, multicomponent intervention, ergonomics, etc.	39 RCTs (31 in MA)	NR
Slemp, 2019^33^	1) Universal 2) Selective	Efficacy of contemplative interventions in reducing psychological distress in employees.	1) General working population from mixed occupations 2) Healthcare (various healthcare professionals)	*Psychosocial:* Contemplative interventions (mindfulness, meditation, acceptance and commitment, and other practices).	119 studies (54 RCTs)	3588
Stratton, 2017^29^	1) Universal 3) Indicated	Evaluate the evidence for the effectiveness and examine the relative efficacy of different types of e-health interventions for employees.	1) General working population from mixed occupations 3) Workers from mixed occupations with symptoms of mental health conditions (eg, depression, stress)	*Psychosocial (delivered through e-health):* e-health mental health interventions (app or web-based) focused on the mental health of employees (CBT, stress management, mindfulness, etc).	23 studies (22 RCTs)	5720
Vega-Escaño, 2020^32^	1) Universal	Evaluate the impact of interventions to improve or reduce insomnia in the workforce through randomised clinical trials.	Workers from mixed occupations	*Psychosocial:* Stress management, CBT, expressive writing, etc.	22 RCTs (12 in MA)	1620
Wasson, 2020^39^	2) Selective	Synthesise the effects of mindfulness-based interventions on self-compassion among healthcare professionals.	Healthcare (various healthcare professionals)	*Psychosocial:* Mindfulness-based interventions.	11 studies (6 RCTs)	349
West, 2016^20^	2) Selective	Interventions to prevent and reduce physician burnout.	Healthcare (physicians)	*Psychosocial:* Small group curricula, stress management and self-care training, communication skills training, belonging intervention, etc.	15 RCTs	716

Whenever possible, the number of participants is based on the randomised trials and on the exact number of participants who were included in the analyses.

Content of interventions: We have specified the delivery mode of the interventions when a meta-analysis was completely focused on only one type of delivery mode (ie, e-health). When the mode of delivery is not specified, it means that different delivery formats could be included in the review (eg, individual, group, e-health).

*Nine were individual-level interventions, and their effects were reported separately.

CBT, cognitive–behavioral therapy; MA, meta-analysis; MH, mental health; NR, not reported; QoL, quality of life; SWB, subjective well-being.

We classified 16 meta-analyses according to the three main groups of interventions, with some of the meta-analyses reporting data for multiple types: (1) universal interventions (n=8 meta-analyses), (2) selective interventions, with all identified reviews focusing on healthcare workers (n=8 meta-analyses) and (3) indicated interventions for workers with symptoms of mental health conditions or disorders (n=5 meta-analyses).

The content or focus of included interventions varied for the main intervention group (universal, selective, indicated). Psychosocial interventions were the most widely examined type in all three groups, with some broader meta-analyses focusing on any type of psychosocial intervention, while some were more specific to a subtype (eg, mindfulness-based^21^) or a particular delivery format (eg, e-health^29^). Physical activity or lifestyle programmes were much less frequently included and were available only for all general workers and for healthcare workers, but not for workers with symptoms of mental health conditions. The interventions were mainly compared with control conditions, involving mostly care-as-usual, waiting list and assessment only. For 14 out of the 16 meta-analyses, these effect sizes were derived exclusively from RCTs, while two meta-analyses reported effect sizes mainly from RCTs (96% and 77%) but included a minority of non-RCTs.

In total, 66 pooled effect sizes were extracted from the meta-analyses, 23 for universal interventions, 29 for selective interventions delivered to healthcare workers and 14 for indicated interventions for workers with symptoms of mental health conditions.

### Quality of the included reviews

**Figure 2:**
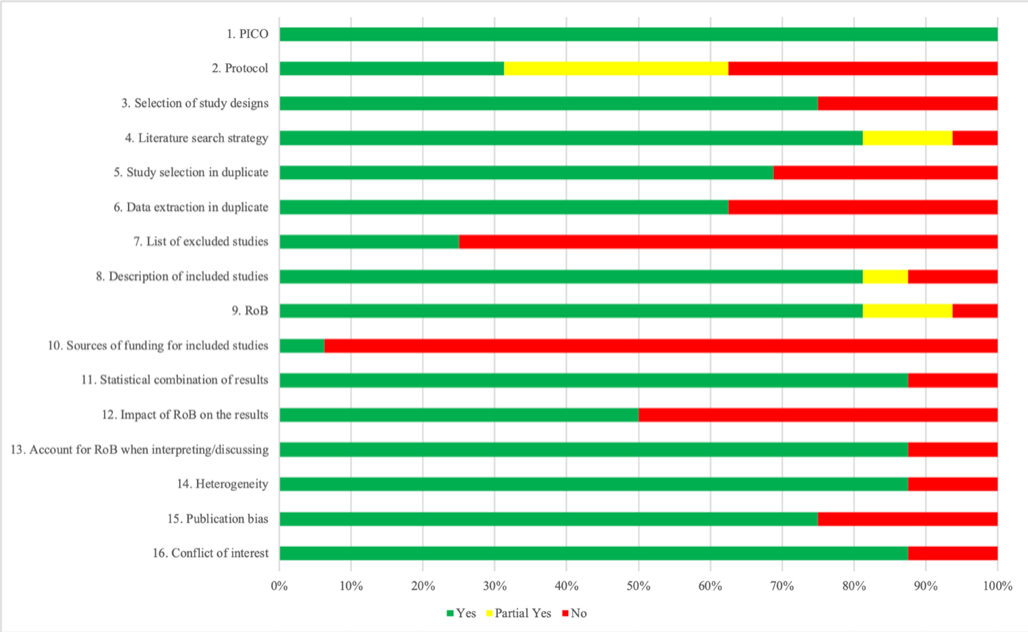
Quality of included meta-analyses based on AMSTAR-2.

The quality of the meta-analyses varied, although most of the AMSTAR-2 items were rated with positive scores (figure 2). All the reviews provided an adequate definition of the PICO (Participants, Interventions, Comparators, Outcomes), and the vast majority (87.5%) used adequate methods for pooling, took RoB into account when interpreting the results, explored heterogeneity and reported conflicts of interest. The majority of the reviews (81.25%) conducted comprehensive searches, described studies in detail and used suitable tools for RoB, and most of them (75%) explained the selection of study designs and explored publication bias. Study selection and data extraction conducted in duplicate was reported for 68.75% and 62.5% of the reviews, respectively. Notably, only half of the meta-analyses statistically examined the influence of RoB on their outcomes. A registered protocol was available for only 31.25% of the reviews, only 25% reported a list of the excluded full texts with reasons and only one explored sources of funding. The AMSTAR-2 ratings for each meta-analysis are presented in online supplemental eTable 1.

### Effects of universal workplace interventions

Eight meta-analyses reported on the effects of universal interventions. Two included psychosocial, physical activity or lifestyle interventions^30 31^ and six focused on psychosocial interventions, such as cognitive–behavioural therapy (CBT)-based interventions, relaxation or stress management programmes.^22 23 29 32–34^ Three meta-analyses focused only on e-health formats,^22 29 34^ while the remaining seven included different types of delivery formats (eg, individual, group, e-health). Available outcomes included symptoms of mental health conditions (n=14), positive mental health (n=6), work-related outcomes (n=2) and quality of life (n=1). A detailed overview of the effects of universal interventions is presented in table 2, along with the GRADE assessments for each outcome. Most of the outcomes were rated as low (12/23) or as very low certainty (8/23), and only 3 were rated as moderate or high.

**Table 2:** Effects of *universal* interventions for supporting mental health at the workplace

Subtypes of interventions	Outcome domain	Specific outcome	*k*	n	SMD (95% CI)	P value	*I* ^2^ (95% CI)	Certainty*	Source
Psychosocial, physical activity or lifestyle (various types)	Positive MH	SWB	54	NA	0.51 (0.31 to 0.71)	<0.01	87 (84 to 90)	⊕◯◯◯	Sakuraya, 2020^30^
	QoL and Funct.	QoL	9	NA	0.77 (0.28 to 1.26)	0.02	82 (67 to 90)	⊕◯◯◯	
Physical activity or lifestyle (various types)	Work related	Work ability	9	1502	0.12 (0.01 to 0.22)	0.03	8 (0 to 68)	⊕⊕⊕◯	Oakman, 2018^31^
Physical activity	Positive MH	SWB	12	NA	0.58 (NA)	0.1	92 (88 to 95)	⊕◯◯◯	Sakuraya, 2020^30^
Psychosocial (various types)	MH symptoms	Burnout (Exhaust.)	25	NA	0.17 (0.03 to 0.32)	<0.05	NA	⊕◯◯◯	Maricuţoiu, 2016^23^
		Burnout (Depers.)	23	NA	−0.008 (−0.15 to 0.13)	ns	NA	⊕◯◯◯	
		Burnout (Pers. Acc.)	23	NA	0.1 (−0.07 to 0.28)	ns	NA	⊕◯◯◯	
		Insomnia	14	1620	MD (ISI): 2.21 (1.06 to 3.36)	0.0002	88 (82 to 92)	⊕◯◯◯	Vega-Escaño, 2020^32^
Psychosocial (mindfulness and contemplative)	MH symptoms	General distress	16	1161	0.49 (0.32 to 0.66)	NA	70 (50 to 82)	⊕⊕◯◯	Slemp, 2019^33^
	Positive MH	SWB	13	NA	0.86 (0.19 to 1.53)	0.03	90 (85 to 94)	⊕◯◯◯	Sakuraya, 2020^30^
Psychosocial (CBT-based)	Positive MH	SWB	11	NA	0.22 (0.04 to 0.40)	0.03	70 (44 to 84)	⊕◯◯◯	Sakuraya, 2020^30^
Psychosocial (various types, delivered through e-health)	MH symptoms	Stress, depression, psychosocial distress	12	2954	0.25 (0.11 to 0.40)	<0.001	71 (47 to 84)	⊕⊕⊕◯	Carolan, 2017^22^
		Stress	13	NA	0.27 (0.15 to 0.39)	<0.001	25 (NA)	⊕⊕◯◯	Phillips, 2019^33^
		Depression	6	NA	0.2 (0.03 to 0.35)	<0.05	41 (NA)	⊕⊕◯◯	
		Anxiety	6	NA	0.2 (−0.04 to 0.44)	ns	59 (NA)	⊕◯◯◯	
		Burnout—overall	2	NA	0.26 (0.02 to 0.50)	<0.05	0 (NA)	⊕⊕◯◯	
		Insomnia	2	NA	0.45 (−0.16 to 1.06)	ns	59 (NA)	⊕◯◯◯	
	Positive MH	Well-being	7	NA	0.35 (0.25 to 0.46)	<0.001	0 (NA)	⊕⊕◯◯	
		Mindfulness	5	NA	0.42 (0.24 to 0.6)	<0.001	0 (NA)	⊕⊕◯◯	
	Work related	Work effectiveness	6	1116	0.18 (0.06 to 0.30)	0.003	0 (0 to 75)	⊕⊕⊕⊕	Carolan, 2017^22^
Psychosocial (mindfulness and contemplative, delivered through e-health)	MH symptoms	Depression, anxiety, stress	6	414	0.6 (0.34 to 0.85)	<0.001	0 (0 to 75)	⊕⊕◯◯	Stratton, 2017^29^
Psychosocial (CBT, delivered through e-health)	MH symptoms	Depression, anxiety, stress	6	1733	0.15 (0.05 to 0.26)	0.001	13 (NA)	⊕⊕◯◯	Stratton, 2017^29^
Psychosocial (stress management, delivered through e-health)	MH symptoms	Depression, anxiety, stress	4	1189	−0.03 (−0.16 to 0.09)	0.57	0 (NA)	⊕◯◯◯	Stratton, 2017^29^

*k* is based on the number of comparisons between interventions and control conditions included in the analyses.

Subtypes of interventions: This column specifies the major intervention categories (psychosocial, physical activity, lifestyle) as well as the content of the interventions included (eg, CBT-based, mindfulness, etc). When a meta-analysis included various interventions with different contents pooled together, we specified it as ‘various types’. When we specified ‘delivered through e-health’, it meant that all interventions included in a meta-analysis were delivered through e-health technologies.

*Certainty levels: ⊕◯◯◯=very low, ⊕⊕◯◯=low, ⊕⊕⊕◯=moderate, ⊕⊕⊕⊕=high.

CBT, cognitive–behavioural therapy; Depers., depersonalisation; Exhaust., exhaustion; Funct., functioning; ISI, Insomnia Severity Index; *k*, number of studies or comparisons included in the analysis; MD, mean difference; MH, mental health; n, number of participants in the analysis; NA, not reported in the review or not possible to impute or calculate; ns, not significant; Pers. Acc., personal accomplishment; QoL, quality of life; SMD, standardised mean difference; SWB, subjective well-being.

Overall, when pooled together in the same analysis, psychosocial, physical activity and lifestyle interventions were associated with moderate and large effects on positive mental health (*well-being*: SMD=0.51, 95% CI 0.31 to 0.71) and quality of life (*overall quality of life*: SMD=0.77, 95% CI 0.28 to 1.26) (table 2). A meta-analysis that examined physical activity and lifestyle programmes showed a negligible but significant effect of these interventions on *work ability* (SMD=0.12, 95% CI 0.01 to 0.22), which was rated as moderate certainty based on GRADE.

Different subtypes of psychosocial interventions (eg, CBT, mindfulness, stress management, psychoeducation, problem-solving) delivered using multiple formats (ie, individual, group, e-health) resulted in small effects on symptoms of mental health conditions, specifically *burnout (exhaustion*) (SMD=0.17, 95% CI 0.03 to 0.32) and *insomnia* (MD=−2.21 in the Insomnia Severity Index, 95% CI 1.06 to 3.36). When examining more specific subtypes of psychosocial interventions based on content, mindfulness and contemplative interventions yielded a moderate effect on *general distress* (SMD=0.49, 95% CI 0.32 to 0.66) and a large effect on *subjective well-being* (SMD=0.86, 95% CI 0.19 to 1.53), while CBT-based interventions showed somewhat smaller effects on the latter outcome (SMD=0.22, 95% CI 0.04 to 0.40) (table 2).

Three meta-analyses examined specifically psychosocial interventions delivered through e-health formats, demonstrating small effects on symptoms of mental health conditions, namely *overall symptoms of common mental disorders* (ie, depression, psychological distress, stress) (SMD=0.25, 95% CI 0.11 to 0.40) (moderate certainty based on GRADE), *depression* (SMD=0.20, 95% CI 0.03 to 0.35), *burnout* (SMD=0.26, 95% CI 0.02 to 0.5) and *stress* (SMD=0.27, 95% CI 0.15 to 0.39). Psychosocial interventions delivered through e-health also showed small to medium effects on two positive mental health outcomes, that is, *well-being* (SMD=0.35, 95% CI 0.25 to 0.46) and *mindfulness* (SMD=0.42, 95% CI 0.24 to 0.60), and a small effect on *work effectiveness* (SMD=0.18, 95% CI 0.06 to 0.3), which was the only outcome in this umbrella review that achieved a high level of certainty based on the GRADE assessment.

There was no available evidence for universal interventions on substance use, suicidal behaviours or potential adverse effects.

### Effects of selective workplace interventions

The effects of selective workplace interventions were available in eight meta-analyses, all of which focused on healthcare professionals. One meta-analysis included various psychosocial, physical activity and lifestyle interventions,^35^ and seven meta-analyses focused on different types of psychosocial interventions, with four of them specifically examining mindfulness and contemplative interventions.^20 21 33 36–39^ Most of the reviews focused on nurses and physicians, although one review also included social workers, psychologists and other allied healthcare staff.^36^ All meta-analyses reported on unselected (universal) samples of healthcare workers, except for one meta-analysis^20^ that also reported separate effects for a subgroup of physicians with elevated symptoms of burnout (which should be considered as selective–indicated intervention). Available outcomes included symptoms of mental health conditions (n=16), positive mental health (n=9), work-related outcomes (n=1), quality of life (n=1), adverse effects (n=1) and suicide-related outcomes (n=1).

A detailed overview of the effects of these interventions is presented in table 3, along with the GRADE assessments for each outcome. Most of the outcomes were rated as low (7/29) or very low certainty (15/29), and 7 outcomes were assessed as moderate certainty.

**Table 3:** Effects of *selective* interventions for supporting mental health at the workplace

Subtypes of interventions	Outcome domain	Specific outcome	*k*	n	SMD (95% CI)	P value	*I* ^2^ (95% CI)	Certainty*	Source
Psychosocial, physical activity or lifestyle (various types)	MH symptoms	Stress	7	420	0.6 (NA)	<0.00001	NA	⊕⊕◯◯	Melnyk, 2020^35^
		Anxiety	5	738	0.2 (NA)	0.03	NA	⊕⊕◯◯	
		Depression	3	719	0.13 (NA)	0.001	NA	⊕⊕◯◯	
	Positive MH	Resilience	4	154	0.58 (NA)	0.001	NA	⊕◯◯◯	
		Mindfulness	5	283	0.85 (NA)	<0.00001	NA	⊕◯◯◯	
	QoL and Funct.	QoL	3	98	0.28 (NA)	0.2	NA	⊕◯◯◯	
Psychosocial (various types)	MH symptoms	Anxiety	5	231	0.06 (−0.23 to 0.35)	0.67	0 (0 to 79)	⊕◯◯◯	Kunzler, 2020^36^
		Depression	14	788	0.29 (0.09 to 0.50)	0.005	42 (0 to 69)	⊕◯◯◯	
		Stress	17	997	0.61 (0.15 to 1.07)	0.01	90 (86 to 93)	⊕◯◯◯	
		Burnout (overall)	5	410	MD (% abs. red MBI): 6 (−7 to 19)	0.37	45 (NA)	⊕⊕◯◯	West, 2016^20^
		Burnout (Exhaust.)	12	499	MD (% abs. red MBI): 2.06 (0.27 to 3.86)	0.02	15 (NA)	⊕⊕⊕◯	
		Burnout (Depers.)	11	472	MD (% abs. red MBI): 0.92 (−0.05 to 1.90)	0.06	31 (NA)	⊕⊕⊕◯	
	Positive MH	Well-being or QoL	13	1494	0.14 (−0.01 to 0.30)	0.07	31 (0 to 64)	⊕◯◯◯	Kunzler, 2020^36^
		Optimism	3	169	0.41 (0.10 to 0.72)	0.009	0 (0 to 90)	⊕◯◯◯	
		Self-efficacy	6	461	0.43 (0.25 to 0.62)	<0.00001	0 (0 to 75)	⊕◯◯◯	
		Positive emotions	2	212	0.85 (0.17 to 1.53)	0.01	82 (NA)	⊕◯◯◯	
		Resilience	12	690	0.45 (0.25 to 0.65)	<0.0001	41 (0 to 70)	⊕◯◯◯	
		Active coping	3	137	0.28 (−0.31 to 0.87)	0.35	52 (0 to 86)	⊕◯◯◯	
	Adverse effects	Adverse events	3	784	No potential adverse or undesired effects			⊕⊕◯◯	
	Suicide	Suicidal ideation	1	199	RR=0.40 (0.17 to 0.91)	0.03	NA	⊕◯◯◯	Petrie, 2019^37^
Psychosocial (various types) for healthcare workers with elevated symptoms of burnout (selective–indicated)	MH symptoms	Burnout (Exhaust.)	8	505	MD (% abs. red. MBI): 13.14 (4.70 to 21.58)	0.002	0 (0 to 68)	⊕⊕⊕◯	West, 2016^20^
		Burnout (Depers.)	6	265	MD (% abs. red MBI): −0.41 (−10.08 to 9.25)	0.93	7 (0 to 76)	⊕◯◯◯	
Psychosocial (mindfulness and contemplative interventions)	MH symptoms	Burnout	5	288	0.26 (0.03 to 0.50)	0.03	0 (0 to 76)	⊕⊕⊕◯	Fendel, 2021^21^
		Stress	4	136	0.55 (0.14 to 0.95)	<0.01	24 (0 to 88)	⊕⊕⊕◯	
		General distress	18	849	0.21 (0.04 to 0.38)	NA	42 (0 to 67)	⊕⊕⊕◯	Slemp, 2019^33^
		Anxiety (state)	6	231	0.78 (0.18 to 1.39)	<0.05	77 (NA)	⊕◯◯◯	Guillaumie, 2017^38^
		Depression	4	214	0.51 (0.23 to 0.78)	<0.05	0 (NA)	⊕⊕⊕◯	
	Work related	Work satisfaction	3	67	0.23 (−0.27 to 0.72)	ns	0 (NA)	⊕⊕◯◯	
	Positive MH	Self-compassion	6	349	0.58 (0.19 to 0.97)	NA	56 (0 to 82)	⊕⊕◯◯	Wasson, 2020^39^

All the identified reviews were focused on healthcare workers.

*k* is based on the number of comparisons between interventions and control conditions included in the analyses.

Interventions: This column specifies the major intervention categories (psychosocial, physical activity, lifestyle) as well as the content of the interventions included (eg, CBT-based, mindfulness, etc). When a meta-analysis included various interventions with different contents pooled together, we specified it as ‘various types’. When we specified ‘delivered through e-health’ it meant that all interventions included in a meta-analysis were delivered through e-health technologies.

*Certainty levels: ⊕◯◯◯=very low, ⊕⊕◯◯=low, ⊕⊕⊕◯=moderate, ⊕⊕⊕⊕=high.

abs. red., absolute reduction; Depers., depersonalisation; Exhaust., emotional exhaustion; Funct., functioning; *k*, number of studies or comparisons included in the analysis; MBI, Maslach Burnout Inventory; MD, mean difference; MH, mental health; n, number of participants in the analysis; NA, not reported in the review or not possible to impute or calculate; ns, not significant; QoL, quality of life; RR, risk ratio; SMD, standarised mean difference.

When all types of selective interventions (psychosocial, physical activity and lifestyle) were pooled together in the same analyses, they were associated with small and moderate effects on symptoms of mental health conditions, that is, *anxiety* (SMD=0.20, p=0.03) and *stress* (SMD=0.60, p<0.0001), as well as moderate to large effects on positive mental health symptoms, that is, *resilience* (SMD=0.58, p=0.001) and *mindfulness* (SMD=0.85, p<0.0001) on health workers (nurses and physicians) (table 3).

Focusing on psychosocial interventions, different subtypes pooled together (eg, CBT, resilience training, mindfulness-based, communication skills) showed a small and moderate effect on symptoms of mental health conditions, namely *depression* (SMD=0.29, 95% CI 0.09 to 0.50) and *stress* (SMD=0.61, 95% CI 0.15 to 1.07). Moreover, such interventions had an absolute reduction of 2.06% on *burnout* symptoms (emotional exhaustion) in physicians, reaching a 13.14% reduction when these interventions were administered to the subgroup of physicians that showed heightened symptoms of burnout at baseline (moderate certainty based on GRADE). Regarding positive mental health, psychosocial interventions yielded small to moderate effects on *optimism* (SMD=0.41, 95% CI 0.1 to 0.72), *self-efficacy* (SMD=0.43, 95% CI 0.25 to 0.62) and *resilience* (SMD=0.45, 95% CI 0.25 to 0.65), and a large effect on *positive emotions* (SMD=0.85, 95% CI 0.17 to 1.53). No potential *adverse or undesired effects* were observed for these interventions.

Four meta-analyses focused specifically on the effects of mindfulness-based interventions, which resulted in a wide range of effects on symptoms of mental health conditions (table 3), ranging from small on *general distress* (SMD=0.21, 95% CI 0.04 to 0.38) and *burnout* (SMD=0.26, 95% CI 0.03 to 0.50) (both with moderate certainty), to moderate and large effects on *depression* (SMD=0.51, 95% CI 0.23 to 0.78) (moderate certainty), *stress* (SMD=0.55, 95% CI 0.14 to 0.95) (moderate certainty) and *anxiety* (SMD=0.78, 95% CI 0.18 to 1.39). Mindfulness-based interventions also showed a moderate improvement in a positive mental health outcome, *self-compassion* (SMD=0.58, 95% CI 0.19 to 0.97). Work-related outcomes were only examined in the context of mindfulness-based interventions, resulting in a non-significant effect size on *work satisfaction* (SMD=0.23, 95% CI −0.27 to 0.72).

Finally, one review reported the effects of a web-based CBT intervention on suicide-related outcomes based on the results of one RCT, showing that physicians who followed the e-health programme were 60% less likely to report *suicidal ideation* than the attention-control group (risk ratio=0.40, 95% CI 0.17 to 0.91).

There was no available evidence for selective interventions on substance use outcomes and on other at-risk groups of workers, and there was very limited evidence for adverse effects, suicide-related outcomes and work-related outcomes.

### Effects of indicated workplace interventions

The effects of workplace interventions delivered to workers with symptoms of mental disorders were retrieved from five meta-analyses.^22 23 29 34 40^ All of these meta-analyses examined the effects of psychosocial interventions on symptoms of mental health conditions (n=12) and work-related outcomes (n=1).

A detailed overview of the effects of interventions for this target group is presented in table 4, along with the GRADE assessments for each outcome. Most of the outcomes were evaluated as very low (10/14) or low certainty (2/14), and 2 were rated as moderate certainty.

**Table 4:** Effects of *indicated* interventions for supporting mental health at the workplace

Subtypes of interventions	Outcome domain	Specific outcome	*k*	n	SMD (95% CI)	P value	*I* ^2^ (95% CI)	Certainty*	Source
Psychosocial (various types)	MH symptoms	Burnout (Exhaust.)	5	NA	0.26 (−0.01 to 0.52)	ns	NA	⊕◯◯◯	Maricuţoiu, 2016^23^
		Burnout (Depers.)	5	NA	0.37 (−0.19 to 0.93)	ns	NA	⊕◯◯◯	
		Burnout (Pers. Acc.)	4	NA	−0.44 (−1.08 to 0.20)	ns	NA	⊕◯◯◯	
		Depression	16	4258	0.4 (0.25 to 0.54)	NA	62 (NA)	⊕⊕◯◯	Nigatu, 2019^40^
Psychosocial (CBT-based)	MH symptoms	Depression	10	3134	0.44 (0.26 to 0.61)	NA	62 (NA)	⊕⊕⊕◯	Nigatu, 2019^40^
Psychosocial (various types, delivered through e-health)	MH symptoms	Stress, depression, psychological distress	9	1844	0.52 (0.28 to 0.75)	<0.001	83 (69 to 91)	⊕◯◯◯	Carolan, 2017^22^
		Stress	9	NA	0.84 (0.55 to 1.13)	<0.001	87 (NA)	⊕◯◯◯	Phillips, 2019^34^
		Depression	11	NA	0.4 (0.21 to 0.53)	<0.001	62 (NA)	⊕◯◯◯	
		Anxiety	9	NA	0.42 (0.23 to 0.61)	<0.001	70 (NA)	⊕◯◯◯	
		Burnout (overall)	6	NA	0.6 (0.3 to 0.88)	<0.001	81 (NA)	⊕◯◯◯	
		Insomnia	5	NA	0.8 (0.22 to 1.39)	<0.01	95 (NA)	⊕◯◯◯	
	Work related	Work effectiveness	7	1465	0.32 (0.04 to 0.61)	0.03	87 (74 to 93)	⊕⊕◯◯	Carolan, 2017^22^
Psychosocial (CBT-based, delivered through e-health)	MH symptoms	Depression, anxiety, stress	5	914	0.13 (−0.01 to 0.27)	0.06	7 (NA)	⊕⊕⊕◯	Stratton, 2017^29^
Psychosocial (stress management, delivered through e-health)	MH symptoms	Depression, anxiety, stress	2	414	0.64 (0.43 to 0.85)	0.001	70 (NA)	⊕◯◯◯	Stratton, 2017^30^

*k* is based on the number of comparisons between interventions and control conditions included in the analyses.

Subtypes of interventions: This column specifies the major intervention categories (psychosocial, physical activity, lifestyle) as well as the content of the interventions included (eg, CBT-based, mindfulness, etc). When a meta-analysis included various interventions with different contents pooled together, we specified it as ‘various types’. When we specified ‘delivered through e-health’, it meant that all interventions included in a meta-analysis were delivered through e-health technologies.

*Certainty levels: ⊕◯◯◯=very low, ⊕⊕◯◯=low, ⊕⊕⊕◯=moderate, ⊕⊕⊕⊕=high.

Depers., depersonalisation; Exhaust., exhaustion; Funct., functioning; *k*, number of studies or comparisons included in the analysis; MD, mean difference; MH, mental health; n, number of participants in the analysis; NA, not reported in the review or not possible to impute or calculate; ns, not significant; QoL, quality of life; SMD, standarised mean difference.

In employees with elevated symptoms of depression, different subtypes of psychosocial interventions pooled showed small to moderate effects in *depressive symptomatology* (SMD=0.40, 95% CI 0.25 to 0.54) (table 4). Similar effects on *depressive symptoms* were found specifically for CBT-based interventions (SMD=0.44, 95% CI 0.26 to 0.61) (moderate certainty). In workers with elevated symptoms of burnout, there was no evidence of a difference between psychosocial interventions and control conditions in reducing any symptoms related to *burnout*.

Two meta-analyses specifically focused on the effectiveness of psychosocial interventions administered through e-health platforms in workers presenting symptoms of common mental disorders (eg, elevated stress, depression, insomnia) (table 4). E-health interventions showed small to moderate effects on *depression* (SMD=0.40, 95% CI 0.21 to 0.53), *anxiety* (SMD=0.42, 95% CI 0.23 to 0.61) and on *overall symptoms of common mental disorders* (ie, stress, depression and psychological distress) (SMD=0.52, 95% CI 0.28 to 0.75), moderate effects on *burnout* (SMD=0.60, 95% CI 0.30 to 0.88) and large effects on *stress* (SMD=0.84, 95% CI 0.55 to 1.13) and *insomnia* (SMD=0.80, 95% CI 0.22 to 1.39). E-health interventions were also effective for improving *work effectiveness* (SMD=0.32, 95% CI 0.04 to 0.61), resulting in a small pooled effect size. Regarding specific types of e-health psychosocial interventions, stress management programmes showed moderate effects on *overall symptoms of common mental disorders* (ie, depression, anxiety, stress) (SMD=0.64, 95% CI 0.43 to 0.85).

There was no available evidence for indicated interventions on positive mental health, substance use, suicidal behaviours, quality of life and potential adverse effects. No evidence was found for other types of interventions than psychosocial (ie, lifestyle, physical activity).

## Discussion

In the current umbrella review, we assessed and summarised the most updated evidence of universal, selective and indicated interventions for the protection of mental health at the workplace. By conducting a systematic search, we reviewed 16 meta-analyses synthesising the effects of psychosocial, physical activity and lifestyle interventions on a total of 66 outcomes, including symptoms of mental health conditions, quality of life, positive mental health and work-related outcomes.

For universal interventions, there was some evidence suggesting that physical activity and lifestyle interventions could improve work-related outcomes but with very small effects. Most of the evidence was dedicated to psychosocial interventions (eg, CBT-based, mindfulness, stress management programmes), showing small to moderate effects on positive mental health and on symptoms of mental health conditions, such as burnout, insomnia or general distress. Universal psychosocial interventions delivered through e-health had mostly small effects across various symptoms of mental health conditions and positive mental health outcomes, with many analyses rated as low or very low certainty. We should note that the only high certainty outcome in this review was found for e-health interventions, which resulted in a small effect on work effectiveness. Regarding selective interventions for at-risk workers (ie, healthcare professionals), most of the evidence was also focused on psychosocial interventions, showing small to moderate effects on symptoms of mental health conditions, and somewhat larger effects on positive mental health, with certainty levels ranging from very low to moderate. Finally, for indicated interventions addressed at workers with elevated symptoms of mental health conditions, all the identified evidence was focused on psychosocial interventions. Such interventions showed small effects on depression, with low to moderate certainty. Indicated interventions delivered through e-health platforms were associated with moderate to large effects in a range of symptoms of mental health conditions, such as stress, depression or insomnia, and small effects on work-related outcomes. Certainty levels were very low in almost all of the examined outcomes.

This umbrella review revealed some gaps in our knowledge that had not been examined by the latest meta-analytical literature. First, the effects of workplace interventions on very relevant outcomes were seldom available. Remarkably, work-related outcomes were only examined in three meta-analyses. Although many of these interventions might be designed for preventing or addressing mental health symptoms, analysing their impact on outcomes like productivity, absence or work satisfaction is crucial in this setting. It remains unclear whether these outcomes were not examined in the meta-analyses or whether these were not collected in the trials. Other outcomes that were rarely examined were adverse effects, suicide-related outcomes or substance use (not examined at all). Second, we found that there is very little up-to-date meta-analytical evidence around the effectiveness of physical activity and lifestyle workplace interventions. Nevertheless, previous evidence suggested that physical activity interventions might be effective in reducing the severity of mental health problems,^41 42^ and lifestyle approaches, such as dietary interventions, have also been associated with a reduction of symptoms of common mental disorders.^43^ Mental health promotion through physical activity and lifestyle interventions could be a promising option due to their potential impact on physical health, particularly for workers with sedentary jobs. Finally, another gap identified in this review was that all the recent meta-analyses on selective interventions were focused on healthcare workers. Future meta-analyses should update our knowledge on other at-risk professions, such as humanitarian workers, police and firefighters. It should be noted that by the time this umbrella review is published, more recent meta-analyses might be available in the literature (eg, the meta-analysis by Tan and colleagues^44^ on emergency workers).

Overall, the effects of universal interventions were generally small, which is in line with wider literature on this type of intervention, such as school^45^ or higher education settings.^46^ Nevertheless, the use of effect sizes to evaluate the impact of universal interventions presents some disadvantages. More specifically, observing large changes resulting from the intervention might be difficult, given that a large percentage of the target population might not have symptoms of mental health conditions. Such difficulty is particularly relevant when examining only short-term outcomes, which could preclude the examination of possible incubation effects of the intervention.^47^ As a result, the effects of universal interventions might have been underestimated. Yet, achieving small changes in a large-scale population can have a considerable public health benefit, especially in highly prevalent conditions. A further problem is the definition of prevention. Examining the real extent of prevention of new cases of mental disorders is only possible if samples are assessed for diagnostic status at baseline.^48^ However, none of the meta-analyses examined this, due to this rarely being established in the trials. Prevention research in the workplace should be better supported, given the resources needed to conduct ‘true prevention’ trials (eg, large sample sizes, diagnostic interviews). The benefits of workplace interventions could be more substantial if access to these interventions was increased. E-health platforms, which were frequently examined by the included meta-analyses, have such potential. Outside the workplace, there is extensive evidence on the effects of e-health interventions for treating common mental disorders such as depression^49^ or anxiety.^50^ In the workplace setting, the results from the included meta-analyses suggested that mental health promotion through universal and indicated interventions could be possible through e-health. Although no recent evidence was found for at-risk workers such as healthcare workers, e-health interventions might be a promising strategy for them given their heavy workload and variable working shifts. Overall, across all types of prevention and early intervention strategies, e-health formats have the potential to reduce costs and increase the availability of evidence-based interventions by reducing stigma and reaching populations with limited access to face-to-face interventions, such as workers from low- and middle-income countries/rural communities or home–office employees. Nevertheless, these interventions are usually associated with very high attrition rates, which is one of the biggest challenges of the implementation of internet-based interventions.

The umbrella methodology used in this review allowed us to integrate a large amount of literature, assess it and combine it comprehensively. We included universal, selective and indicated workplace interventions, making our findings representative of three important types of preventative and early intervention strategies. Another strength of this review is the inclusion of a broad scope of outcomes, involving important and meaningful outcomes such as functioning, quality of life, work-related outcomes and positive mental health. This is particularly relevant in this context since it has been suggested that occupational recovery might follow a separate course to symptomatic improvement.^51^ Moreover, positive mental health outcomes, like well-being, have been associated with impacts on work-related outcomes such as performance or productivity.^52 53^

This review has some limitations that should be considered. First, we aimed to identify the most updated evidence by including meta-analyses published in the last 5 years, but we may have missed meta-analyses published prior to this time limit. However, the evidence of older systematic reviews might be outdated and thus less informative due to the exponential growth of trials in this field. Second, we focused on the post-test outcomes of these interventions; thus, future research should examine long-term effects. Another important limitation is that the certainty and quality of the evidence were very low, with only 1 of 66 outcomes assessed as high certainty. It should be noted that blinding of participants and personnel in psychosocial trials is most of the times impossible, and this results in higher risk of bias scores. Another limitation is that heterogeneity was very high in many of the pooled effect sizes. Moreover, only half of the meta-analyses statistically examined the influence of risk of bias on their outcomes. Thus, the summarised effects should be interpreted with caution. Finally, given the nature of this study, the evidence that we presented relies on the decisions made by the meta-analysts. Therefore, alternative classifications and pooling of interventions are plausible and might change the overall summary of the results.

## Conclusions

The workplace provides a promising setting for implementing and disseminating mental health promotion strategies. Different types of universal, selective and indicated interventions are available, with variable effects on a range of outcomes that include not only symptoms of mental health conditions but also meaningful outcomes for the employees and the employers (eg, work effectiveness, resilience, etc). Organisations should offer interventions according to the characteristics of the target population given that general workers and workers at a higher risk for mental disorders may benefit from different types of strategies. E-health platforms are promising in improving the access to evidence-based interventions, reducing stigma and offering flexibility to individuals with irregular working hours. Nevertheless, we should note that the quality and certainty of the evidence on the effectiveness of workplace interventions is very modest. Further high-quality research is warranted, particularly on work-related outcomes and including more types of at-risk professions.
