# Supplementary material for: Universal, selective and indicated interventions for supporting mental health at the workplace: an umbrella review of meta-analyses
Source: Occup Environ Med. 2023 Feb 24;80(4):225–36. doi: 10.1136/oemed-2022-108698 (PMC10086469; doi:10.1136/oemed-2022-108698)
Supplement: Supplementary data [file oemed-2022-108698supp001.pdf]

Supplement

Search strings for PubMed

Search	Query
	Time limit: 2015
#17	(#16 AND #2 AND #3 AND #4) AND (#5 OR #6 OR #7 OR #8 OR #9 OR #10 OR #11)
#16	"Occupational Groups"[Mesh] OR "Occupational Health"[Mesh] OR "enterprise*"[tiab] OR "business*"[tiab] OR "employed"[tiab] OR "employee*"[tiab] OR "employer*"[tiab] OR "employment*"[tiab] OR "informal sector*"[tiab] OR "informal work*"[tiab] OR "laborer*"[tiab] OR "labourer*"[tiab] OR "occupation*"[tiab] OR "personnel*"[tiab] OR "professional"[tiab] OR "professionals"[tiab] OR "staff"[tiab] OR "staffing*"[tiab] OR "vocation"[tiab] OR "vocations"[tiab] OR "worker"[tiab] OR "workers"[tiab] OR "workforce"[tiab] OR "workplace*"[tiab] OR "work place*"[tiab] OR "worksite*"[tiab] OR "work site*"[tiab] OR "aide"[tiab] OR "aides"[tiab] OR "audiologist*"[tiab] OR "ambulance*"[tiab] OR "care assistant*"[tiab] OR "clinician*"[tiab] OR "coastguard*"[tiab] OR "coast guard*"[tiab] OR "dentist*"[tiab] OR "detective*"[tiab] OR "disaster responder*"[tiab] OR "doctor*"[tiab] OR "domestic health care"[tiab] OR "domiciliary care*"[tiab] OR "emergency service*"[tiab] OR "emergency responder*"[tiab] OR "emergency medical"[tiab] OR "firefighter*"[tiab] OR "fire fighter*"[tiab] OR "first responder*"[tiab] OR "general practitioner*"[tiab] OR "health care provider*"[tiab] OR "healthcare provider*"[tiab] OR "health visitor*"[tiab] OR "home care service*"[tiab] OR "humanitarian aid*"[tiab] OR "humanitarian relie*"[tiab] OR "humanitarian service*"[tiab] OR "law enforc*"[tiab] OR "lifeguard*"[tiab] OR "life guard*"[tiab] OR "medical resident*"[tiab] OR "medic"[tiab] OR "medics"[tiab] OR "nurse*"[tiab] OR "nursing"[tiab] OR "midwi*"[tiab] OR "paramedic*"[tiab] OR "policemen"[tiab] OR "policeman"[tiab] OR "police men"[tiab] OR "police man"[tiab] OR "police women"[tiab] OR "police woman"[tiab] OR "police officer*"[tiab] OR "firemen"[tiab] OR "fireman"[tiab] OR "fire men"[tiab] OR "fire man"[tiab] OR "fire women"[tiab] OR "fire woman"[tiab] OR "pharmacis*"[tiab] OR "psychologist*"[tiab] OR "physician*"[tiab] OR "practitioner*"[tiab] OR "relief work*"[tiab] OR "rescuer*"[tiab] OR "rescue work*"[tiab] OR "therapist*"[tiab] OR "veterinaria*"[tiab]
#11	"drop out*"[tiab] OR "dropout*"[tiab]
#10	"Absenteeism"[Mesh] OR "Employment"[Mesh] OR "Job Satisfaction"[MeSH] OR "Sick Leave"[Mesh] OR "Retirement"[Mesh] OR "Work Capacity Evaluation"[Mesh] OR "absenteeism*"[tiab] OR "back to work"[tiab] OR "early retir*"[tiab] OR "effectiveness"[tiab] OR "employabil*"[tiab] OR "employment status*"[tiab] OR "job length*"[tiab] OR "job retenti*"[tiab] OR "job satisf*"[tiab] OR "work satisf*"[tiab] OR "medical certificate*"[tiab] OR "presenteeism*"[tiab] OR "productivit*"[tiab] OR "prolonged work*"[tiab] OR "prolonging work*"[tiab] OR "resignati*"[tiab] OR "return to work"[tiab] OR "sick leav*"[tiab] OR "sickness absen*"[tiab] OR "sickness presen*"[tiab] OR "sick listing*"[tiab] OR "sustainable work*"[tiab] OR "sustained work*"[tiab] OR "unemploy*"[tiab] OR "work absence*"[tiab] OR "work abilit*"[tiab] OR "work capacit*"[tiab] OR "work disabilit*"[tiab] OR "work engag*"[tiab] OR "work function*"[tiab] OR "work participati*"[tiab] OR "work performan*"[tiab] OR "work retention"[tiab] OR "turnover*"[tiab] OR "turn over*"[tiab]
#9	"Quality of Life"[Mesh] OR "Quality-Adjusted Life Years"[Mesh] OR "disability adjusted life"[tiab] OR "qaly"[tiab] OR "daly"[tiab] OR "functioning"[tiab] OR "functional abilit*" OR "functionalit*" OR "hrqol*"[tiab] OR "life activit*"[tiab] OR "life participati*"[tiab] OR "life stress*"[tiab] OR "qol"[tiab] OR "qoli"[tiab] OR "quality of life*"[tiab] OR "self car*"[tiab] OR "selfcar*"[tiab] OR "sickness impact profile*" [tiab] OR "social function*"[tiab] OR "social participati*"[tiab]
#8	"Substance-Related Disorders"[Mesh] OR "alcohol abus*"[tiab] OR "alcohol misus*"[tiab] OR "alcohol us*"[tiab] OR "alcoholis*"[tiab] OR "amphetamin*"[tiab] OR "binge drinking*"[tiab] OR "cannabis*"[tiab] OR "cocain*"[tiab] OR "diacetylmorphin*"[tiab] OR "diamorphin*"[tiab] OR "drug abus*"[tiab] OR "drug misus*"[tiab] OR "drug us*"[tiab] OR "drunk*"[tiab] OR "drinking"[tiab] OR "ecstasy*"[tiab] OR "xtc"[tiab] OR "fentanyl*"[tiab] OR "hashish*"[tiab] OR "heroin*"[tiab] OR "marihuan*"[tiab] OR "marijuan*"[tiab] OR "mdma"[tiab] OR "methadon*"[tiab] OR "methamphetamin*"[tiab] OR "methylenedioxymethamphetamin*"[tiab] OR "morphin*"[tiab] OR "opiate*"[tiab] OR "opioid*"[tiab] OR "thc"[tiab] OR "crystal meth*"[tiab]

#7	"Self-Injurious Behavior"[Mesh] OR "auto mutilat*"[tiab] OR "automutilat*"[tiab] OR "intentional injur*"[tiab] OR "self harm*"[tiab] OR "selfharm*"[tiab] OR "suicid*"[tiab]
#6	"Mental Disorders"[Mesh] OR "Mental Health"[Mesh] OR "Psychology, Industrial"[Mesh] OR "Stress, Psychological"[Mesh] OR "adjustment"[tiab] OR "affective disorder*"[tiab] OR "anxiet*"[tiab] OR "bipolar*"[tiab] OR "burn out*"[tiab] OR "burnout*"[tiab] OR "CMD" [tiab] OR "depressi*"[tiab] OR "eating disorder*"[tiab] OR "mental disorder*"[tiab] OR "mental health*"[tiab] OR "mental illness*"[tiab] OR "mood disorder*"[tiab] OR "obsessive compulsive disorder*"[tiab] OR "ocd"[tiab] OR "panic disorder*"[tiab] OR "phobi*"[tiab] OR "post traumatic*"[tiab] OR "psychiatric diagnos*"[tiab] OR "psychiatric disease*"[tiab] OR "psychiatric disorder*"[tiab] OR "psychiatric illness*"[tiab] OR "psychological disorder*"[tiab] OR "psychos*"[tiab] OR "psychotic*"[tiab] OR "psychological distress*"[tiab] OR "ptsd"[tiab] OR "ptss"[tiab] OR "somatoform disorder*"[tiab] OR "schizophren*"[tiab] OR "stress*"[tiab]
#5	"Optimism"[Mesh] OR "Personal Satisfaction"[Mesh] OR "Self Concept"[Mesh:NoExp] OR "Self Efficacy"[Mesh] OR "Self-Control"[Mesh] OR "life engag*"[tiab] OR "life satisf*"[tiab] OR "meaning of life"[tiab] OR "purpose in life"[tiab] OR "positive affect*"[tiab] OR "positive emotion*"[tiab] OR "resilien*"[tiab] OR "self concept*"[tiab] OR "self control*"[tiab] OR "self efficac*"[tiab] OR "self esteem*"[tiab] OR "swb"[tiab] OR "well being*"[tiab] OR "wellbeing*"[tiab]
#4	"Psychotherapy"[Mesh] OR "Primary Prevention"[Mesh] OR "Secondary Prevention"[Mesh] OR "Tertiary Prevention"[Mesh] OR "Counseling"[Mesh] OR "Social Support"[Mesh] OR "Psychology, Positive"[Mesh] OR "Sports"[Mesh] OR "Healthy Lifestyle"[Mesh] OR "Sedentary Behavior"[Mesh] OR "Acceptance and Commitment Therapy"[Mesh] OR "Nutrition Therapy"[Mesh] OR "Exercise"[Mesh] OR "Mindfulness"[Mesh] OR "Mind Body Therapies"[Mesh] OR "Internet Based Intervention"[Mesh] OR "therap*"[tiab] OR "treatment*"[tiab] OR "program*"[tiab] OR "prevent*"[tiab] OR "educat*"[tiab] OR "training*"[tiab] OR "course*"[tiab] OR "psychotherap*"[tiab] OR "psychoeduc*"[tiab] OR "psycho educ*"[tiab] OR "solution focused"[tiab] OR "stress manag*"[tiab] OR "stress reduc*"[tiab] OR "self monitoring"[tiab] OR "counsel*"[tiab] OR "coaching*"[tiab] OR "personal development*"[tiab] OR "positive psycho*"[tiab] OR "health promoti*"[tiab] OR "physical activit*"[tiab] OR "exercis*"[tiab] OR "motor activit*"[tiab] OR "sport"[tiab] OR "sports"[tiab] OR "fitness"[tiab] OR "yoga"[tiab] OR "aerobic*"[tiab] OR "multicomponent"[tiab] OR "multi component"[tiab] OR "relaxation"[tiab] OR "mindfulness"[tiab] OR "meditati*"[tiab] OR "cbt"[tiab] OR "emotional regulati*"[tiab] OR "emotion regulati*"[tiab] OR "emotion focused"[tiab] OR "problem solving"[tiab] OR "behavioural activation"[tiab] OR "behavioral activation"[tiab] OR "psychodynamic*"[tiab] OR "psychoanaly*"[tiab] OR "psycho dynamic*"[tiab] OR "psycho analy*"[tiab] OR "self directed"[tiab] OR "self help"[tiab]
#3	("Randomized Controlled Trial" [Publication Type] OR random*[tiab] OR pragmatic clinical trial*[tiab] OR practical clinical trial*[tiab] OR non-inferiority trial*[tiab] OR noninferiority trial*[tiab] OR superiority trial*[tiab] OR equivalence clinical trial*[tiab]) NOT (("Animals"[Mesh]) OR "Models, Animal"[Mesh] NOT humans[mh]) NOT (letter[pt] OR comment[pt] OR editorial[pt])
#2	("Meta-Analysis" [Publication Type] OR "Meta-Analysis as Topic"[Mesh] OR metaanaly*[tiab] OR meta-analy*[tiab] or metanaly*[tiab] OR "Systematic Review" [Publication Type] OR systematic[sb] OR "Cochrane Database Syst Rev"[Journal] or prisma[tiab] OR preferred reporting items[tiab] OR prospero[tiab] OR ((systemati*[ti] OR umbrella[ti] OR structured literature[ti]) AND (review*[ti] OR overview*[ti])) OR systematic review*[tiab] OR umbrella review*[tiab] OR structured literature review*[tiab] OR systematic qualitative review*[tiab] OR systematic quantitative review*[tiab] OR systematic search and review[tiab] OR systematized review[tiab] OR systematised review[tiab] OR systemic review[tiab] OR systematic literature review*[tiab] OR systematic integrative literature review*[tiab] OR systematically review*[tiab] OR scoping literature review*[tiab] OR systematic critical review[tiab] OR systematic integrative review*[tiab] OR systematic evidence review[tiab] OR systematic integrative literature review*[tiab] OR systematic mixed studies review*[tiab] OR systematized literature review*[tiab] OR systematic overview*[tiab] OR Systematic narrative review*[tiab] OR ((systemati*[tiab] OR literature[tiab] OR database*[tiab] OR data-base*[tiab] OR structured[tiab] OR comprehensive*[tiab] OR systemic*[tiab]) AND search*[tiab]) OR (Literature[ti] AND review[ti] AND (database*[tiab] OR data-base*[tiab] OR search*[tiab])) OR ((data extraction[tiab] OR data source*[tiab]) AND study selection[tiab]) OR (search strategy[tiab] AND selection criteria[tiab]) OR (data source*[tiab] AND data synthesis[tiab]) OR

	medline[tiab] OR pubmed[tiab] OR embase[tiab] OR Cochrane[tiab] OR ((critical[ti] OR rapid[ti]) AND (review*[ti] OR overview*[ti] OR synthes*[ti])) OR (((critical*[tiab] OR rapid*[tiab]) AND (review*[tiab] OR overview*[tiab] OR synthes*[tiab]) AND (search*[tiab] OR database*[tiab] OR data-base*[tiab]))) OR metasynthes*[tiab] OR meta-synthes*[tiab]) NOT ("Comment" [Publication Type] OR "Letter" [Publication Type] OR "Editorial" [Publication Type] OR (("Animals"[Mesh] OR "Models, Animal"[Mesh]) NOT "Humans"[Mesh]))
#1	"Occupational Groups"[Mesh] OR "Occupational Health"[Mesh] OR "enterprise*"[ti] OR "business*"[ti] OR "employed"[ti] OR "employee*"[ti] OR "employer*"[ti] OR "employment*"[ti] OR "informal sector*"[ti] OR "informal work*"[ti] OR "laborer*"[ti] OR "labourer*"[ti] OR "occupation*"[ti] OR "personnel*"[ti] OR "professional"[ti] OR "professionals"[ti] OR "staff"[ti] OR "staffing*"[ti] OR "vocation"[ti] OR "vocations"[ti] OR "worker"[ti] OR "workers"[ti] OR "workforce"[ti] OR "workplace*"[ti] OR "work place*"[ti] OR "worksite*"[ti] OR "work site*"[ti] OR "aide"[ti] OR "aides"[ti] OR "audiologist*"[ti] OR "ambulance*"[ti] OR "care assistant*"[ti] OR "clinician*"[ti] OR "coastguard*"[ti] OR "coast guard*"[ti] OR "dentist*"[ti] OR "detective*"[ti] OR "disaster responder*"[ti] OR "doctor*"[ti] OR "domestic health care"[ti] OR "domiciliary care*"[ti] OR "emergency service*"[ti] OR "emergency responder*"[ti] OR "emergency medical"[ti] OR "firefighter*"[ti] OR "fire fighter*"[ti] OR "first responder*"[ti] OR "general practitioner*"[ti] OR "health care provider*"[ti] OR "healthcare provider*"[ti] OR "health visitor*"[ti] OR "home care service*"[ti] OR "humanitarian aid*"[ti] OR "humanitarian relie*"[ti] OR "humanitarian service*"[ti] OR "law enforc*"[ti] OR "lifeguard*"[ti] OR "life guard*"[ti] OR "medical resident*"[ti] OR "medic"[ti] OR "medics"[ti] OR "nurse*"[ti] OR "nursing"[ti] OR "midwi*"[ti] OR "paramedic*"[ti] OR "policemen"[ti] OR "policeman"[ti] OR "police men"[ti] OR "police man"[ti] OR "police women"[ti] OR "police woman"[ti] OR "police officer*"[ti] OR "firemen"[ti] OR "fireman"[ti] OR "fire men"[ti] OR "fire man"[ti] OR "fire women"[ti] OR "fire woman"[ti] OR "pharmacis*"[ti] OR "psychologist*"[ti] OR "physician*"[ti] OR "practitioner*"[ti] OR "relief work*"[ti] OR "rescuer*"[ti] OR "rescue work*"[ti] OR "therapist*"[ti] OR "veterinaria*"[ti] OR "enterprise*"[ot] OR "business*"[ot] OR "employed"[ot] OR "employee*"[ot] OR "employer*"[ot] OR "employment*"[ot] OR "informal sector*"[ot] OR "informal work*"[ot] OR "laborer*"[ot] OR "labourer*"[ot] OR "occupation*"[ot] OR "personnel*"[ot] OR "professional"[ot] OR "professionals"[ot] OR "staff"[ot] OR "staffing*"[ot] OR "vocation"[ot] OR "vocations"[ot] OR "worker"[ot] OR "workers"[ot] OR "workforce"[ot] OR "workplace*"[ot] OR "work place*"[ot] OR "worksite*"[ot] OR "work site*"[ot] OR "aide"[ot] OR "aides"[ot] OR "audiologist*"[ot] OR "ambulance*"[ot] OR "care assistant*"[ot] OR "clinician*"[ot] OR "coastguard*"[ot] OR "coast guard*"[ot] OR "dentist*"[ot] OR "detective*"[ot] OR "disaster responder*"[ot] OR "doctor*"[ot] OR "domestic health care"[ot] OR "domiciliary care*"[ot] OR "emergency service*"[ot] OR "emergency responder*"[ot] OR "emergency medical"[ot] OR "firefighter*"[ot] OR "fire fighter*"[ot] OR "first responder*"[ot] OR "general practitioner*"[ot] OR "health care provider*"[ot] OR "healthcare provider*"[ot] OR "health visitor*"[ot] OR "home care service*"[ot] OR "humanitarian aid*"[ot] OR "humanitarian relie*"[ot] OR "humanitarian service*"[ot] OR "law enforc*"[ot] OR "lifeguard*"[ot] OR "life guard*"[ot] OR "medical resident*"[ot] OR "medic"[ot] OR "medics"[ot] OR "nurse*"[ot] OR "nursing"[ot] OR "midwi*"[ot] OR "paramedic*"[ot] OR "policemen"[ot] OR "policeman"[ot] OR "police men"[ot] OR "police man"[ot] OR "police women"[ot] OR "police woman"[ot] OR "police officer*"[ot] OR "firemen"[ot] OR "fireman"[ot] OR "fire men"[ot] OR "fire man"[ot] OR "fire women"[ot] OR "fire woman"[ot] OR "pharmacis*"[ot] OR "psychologist*"[ot] OR "physician*"[ot] OR "practitioner*"[ot] OR "relief work*"[ot] OR "rescuer*"[ot] OR "rescue work*"[ot] OR "therapist*"[ot] OR "veterinaria*"[ot]

Classification of outcomes for each intervention target

Universal interventions

Critical:

- Mental health symptoms and disorders
- Positive mental health
- Quality of life and functioning
- Work-related outcomes

Important:

- Adverse effects
- Substance use
- Suicide

Selective and Indicated interventions

Critical:

- Mental health symptoms and disorders
- Positive mental health
- Quality of life and functioning
- Substance use
- Suicide
- Work-related outcomes

Important:

- Adverse effects

AMSTAR-2 Quality Assessment

The quality of the included meta-analyses was assessed using AMSTAR-2 (Shea et al., 2017). AMSTAR-2 critically appraises core methodological characteristics of systematic reviews including (1) adequate definition of participants, intervention, comparator, outcome (i.e., PICO), (2) methods were established before the conduct of the review, (3) justification for the study designs, (4) comprehensiveness of the search, (5) study selection and (6) data extraction conducted by two reviewers, (7) list of excluded studies with reasons, (8) detailed description of included studies, (9) adequate methods for Risk of bias assessment (RoB), (10) investigation of sources of funding, (11) appropriate methods for pooling, (12) examination of the impact of RoB on the outcomes, (13) discussion of the impact of RoB in the interpretation of results, (14) adequate exploration of heterogeneity, (15) assessment of publication bias, (16) reporting potential conflict of interests for the review. Items 1, 3, 5, 6, 10, 11, 12, 13, 14, 15 and 16 were rated as positive (Yes) or negative (No), and Items 2, 4, 7, 8, and 9 also included a partially positive answer (Partial Yes). Each item was rated by two independent reviewers (CM, AA), and disagreements were solved by discussion or consultation with a third reviewer (PC).

GRADE Assessment

Further details regarding the ratings of each of the GRADE main factors:

Risk of bias (RoB): We extracted the RoB ratings from the individual studies included in the meta-analyses (when available). We calculated the percentage of trials rated at low, high, and unclear risk of bias. Based on this information, and in order to take consistent decisions across the available evidence, we rated the RoB GRADE item using a decision tree (Figure 1).

Inconsistency: We judged inconsistency by examining heterogeneity statistics (I^2, which indicates the percentage of heterogeneity between effect sizes). Whenever possible, we computed the 95% CI of I^2 and used it in our judgements. We judged inconsistency as serious when I^2 was over than 75% and its 95% CI substantially overlapped with the category of considerable heterogeneity (above 75%). Substantial overlap was estimated with the median of the 95% CI. When the 95% CI could not be calculated, we rated it as serious if heterogeneity was larger than 50% (category of substantial heterogeneity). When I^2 was not reported and could not be calculated, we rated it as serious.

Indirectness: Direct evidence is derived from research that directly compares the interventions which we are interested in, delivered to the participants in which we are interested, and that measures the outcomes important to patients. We rated for each particular comparison how indirect was the reviewed evidence in terms of population, intervention, and outcomes.

Imprecision: We rated this item based on a standard power calculation (α 0.05and β 0.20) for detecting an effect size of 0.2, which requires a sample size of 400 participants in total. We judged as serious all analyses including less than 400 participants. Analyses including less than 100 participants were rated as very serious. We rated it as serious when the number of participants included in the analyses was not available.

Other considerations: For this item we focused on publication bias. We rated it as serious when there was evidence for publication bias in the meta-analyses, based on statistical tests. However, we did not downgrade the evidence when the meta-analyses did not investigate it.

- No data available for risk of bias → serious

- When majority (≥ 60%) of trials are low risk → not serious
- When low risk is between 50-59%:
  - High risk < 25% → not serious
  - High risk ≥ 25% → serious

- When majority (≥ 60%) is high risk → very serious
- When high risk is between 50-59%:
  - Low risk < 25% → very serious
  - Low risk ≥ 25% → serious

- When majority is unclear risk (≥ 60%) → serious
- When unclear risk is between 50-59%:
  - High risk <25% → not serious
  - High risk ≥ 25% → serious

- If both unclear/low risk are < 60%:
  - High risk < 25% → not serious
  - High risk ≥ 25% → serious

Figure 1. Developed decision tree for the assessment of risk of bias in GRADE.

References of the included meta-analyses

Carolan, S., Harris, P. R., & Cavanagh, K. (2017). Improving Employee Well-Being and Effectiveness: Systematic Review and Meta-Analysis of Web-Based Psychological Interventions Delivered in the Workplace. *J Med Internet Res, 19*(7), e271.

Fendel, J. C., Bürkle, J. J., & Göritz, A. S. (2021). Mindfulness-based interventions to reduce burnout and stress in physicians: a systematic review and meta-analysis. *Academic Medicine*, *96*(5), 751-764.

Guillaumie, L., Boiral, O., & Champagne, J. (2017, May). A mixed-methods systematic review of the effects of mindfulness on nurses. *J Adv Nurs, 73*(5), 1017-1034.

Kunzler, A. M., Helmreich, I., Chmitorz, A., KÃ¶nig, J., Binder, H., Wessa, M., & Lieb, K. (2020). Psychological interventions to foster resilience in healthcare professionals. *Cochrane Database Syst Rev, 7*, Cd012527.

Maricuţoiu, L. P., Sava, F. A., & Butta, O. (2016). The effectiveness of controlled interventions on employees' burnout: A meta-analysis. *Journal of Occupational and Organizational Psychology, 89*(1), 1-27.

Melnyk, B. M., Kelly, S. A., Stephens, J., Dhakal, K., McGovern, C., Tucker, S., Hoying, J., McRae, K., Ault, S., Spurlock, E., & Bird, S. B. (2020). Interventions to Improve Mental Health, Well-Being, Physical Health, and Lifestyle Behaviors in Physicians and Nurses: A Systematic Review. *Am J Health Promot, 34*(8), 929-941.

Nigatu, Y. T., Huang, J., Rao, S., Gillis, K., Merali, Z., & Wang, J. (2019). Indicated prevention interventions in the workplace for depressive symptoms: a systematic review and meta-analysis. *American Journal of Preventive Medicine*, *56*(1), e23-e33.

Oakman, J., Neupane, S., Proper, K. I., Kinsman, N., & Nygard, C. H. (2018). Workplace interventions to improve work ability: A systematic review and meta-analysis of their effectiveness. *Scand J Work Environ Health, 44*(2), 134-146.

Petrie, K., Crawford, J., Baker, S. T. E., Dean, K., Robinson, J., Veness, B. G., Randall, J., McGorry, P., Christensen, H., & Harvey, S. B. (2019). Interventions to reduce symptoms of common mental disorders and suicidal ideation in physicians: a systematic review and meta-analysis. *Lancet Psychiatry, 6*(3), 225-234.

Phillips, E. A., Gordeev, V. S., & Schreygg, J. (2019). Effectiveness of occupational e-mental health interventions: a systematic review and meta-analysis of randomized controlled trials. *Scand J Work Environ Health, 45*(6), 560-576.

Sakuraya, A., Imamura, K., Watanabe, K., Asai, Y., Ando, E., Eguchi, H., Nishida, N., Kobayashi, Y., Arima, H., Iwanaga, M., Otsuka, Y., Sasaki, N., Inoue, A., Inoue, R., Tsuno, K., Hino, A., Shimazu, A., Tsutsumi, A., & Kawakami, N. (2020). What Kind of Intervention Is Effective

for Improving Subjective Well-Being Among Workers? A Systematic Review and Meta-Analysis of Randomized Controlled Trials. *Front Psychol, 11*, 528656.

Slemp, G. R., Jach, H. K., Chia, A., Loton, D. J., & Kern, M. L. (2019). Contemplative interventions and employee distress: A meta-analysis. *Stress Health, 35*(3), 227-255.

Stratton, E., Lampit, A., Choi, I., Calvo, R. A., Harvey, S. B., & Glozier, N. (2017). Effectiveness of eHealth interventions for reducing mental health conditions in employees: A systematic review and meta-analysis. *PLoS One, 12*(12), e0189904.

Vega-Escaño, J., Porcel-Gálvez, A. M., Diego-Cordero, R., Romero-Sánchez, J. M., Romero-Saldaña, M., & Barrientos-Trigo, S. (2020). Insomnia Interventions in the Workplace: A Systematic Review and Meta-Analysis. *Int J Environ Res Public Health, 17*(17).

Wasson, R. S., Barratt, C., & O'Brien, W. H. (2020). Effects of Mindfulness-Based Interventions on Self-compassion in Health Care Professionals: a Meta-analysis. *Mindfulness (N Y)*, 1-21.

West, C. P., Dyrbye, L. N., Erwin, P. J., & Shanafelt, T. D. (2016). Interventions to prevent and reduce physician burnout: a systematic review and meta-analysis. *Lancet, 388*(10057), 2272-2281.

eTable 1. AMSTAR-2 ratings of included meta-analyses

**Study**	1	2	3	4	5	6	7	8	9	10	11	12	13	14	15	16
Carolan, 2017	Y	Y	Y	Y	N	N	N	Y	Y	N	Y	Y	Y	Y	Y	Y
Fendel, 2021	Y	Y	Y	Y	Y	Y	Y	Y	Y	Y	Y	Y	Y	Y	Y	Y
Guillaumie, 2017	Y	N	N	PY	N	Y	N	Y	N	N	Y	N	N	Y	Y	N
Kunzler, 2020	Y	PY	Y	PY	N	Y	N	N	PY	N	N	N	Y	N	Y	Y
Maricuţoiu, 2016	Y	PY	Y	PY	Y	Y	N	Y	Y	N	N	N	Y	N	N	N
Melnyk, 2020	Y	N	Y	PY	Y	N	N	Y	Y	N	Y	N	Y	Y	N	Y
Nigatu, 2019	Y	N	Y	PY	N	N	Y	Y	Y	N	Y	Y	Y	Y	Y	Y
Oakman, 2018	Y	Y	N	PY	Y	N	N	Y	Y	N	Y	Y	Y	Y	Y	Y
Petrie, 2019	Y	PY	Y	PY	Y	Y	Y	Y	Y	N	Y	Y	Y	Y	Y	Y
Phillips, 2019	Y	N	Y	PY	N	Y	N	Y	Y	N	Y	Y	Y	Y	Y	Y
Sakuraya, 2020	Y	N	Y	PY	Y	N	N	Y	Y	N	Y	Y	Y	Y	Y	Y
Slemp, 2019	Y	PY	Y	PY	Y	Y	N	PY	Y	N	Y	Y	Y	Y	N	Y
Stratton, 2017	Y	Y	Y	PY	Y	Y	N	Y	PY	N	Y	N	Y	Y	Y	Y
Vega-Escaño, 2020	Y	N	N	PY	Y	Y	N	Y	Y	N	Y	N	Y	Y	Y	Y
Wasson, 2020	Y	PY	Y	N	Y	N	N	N	Y	N	Y	N	N	Y	N	Y
West, 2016	Y	Y	N	PY	Y	Y	Y	Y	Y	N	Y	N	Y	Y	Y	Y

AMSTAR-2 items: (1) adequate definition of PICO, (2) methods established before the review, (3) an explanation for the selection of study design to be included, (4) comprehensiveness of the search strategy, (5) study selection by at least two reviewers, (6) data extraction by at least two reviewers, (7) list of excluded studies with reasons, (8) detailed description of included studies, (9) assessment of risk of bias in included studies, (10) reported sources of funding for the included studies, (11) appropriate methods for pooling results, (12) assessment of the impact of risk of bias on the outcomes, (13) discussion of the impact of risk of bias on results, (14) sources of heterogeneity are explored, (15) assessment of publication bias, (16) reporting potential conflict of interest for the review.
